# The Effect of Anthocyanin-Rich Foods or Extracts on Vascular Function in Adults: A Systematic Review and Meta-Analysis of Randomised Controlled Trials

**DOI:** 10.3390/nu9080908

**Published:** 2017-08-20

**Authors:** Lucy Fairlie-Jones, Kade Davison, Emilie Fromentin, Alison M. Hill

**Affiliations:** 1 School of Pharmacy & Medical Sciences, University of South Australia, Adelaide 5000, Australia; failc001@mymail.unisa.edu.au; 2 School of Health Sciences, University of South Australia, Adelaide 5000, Australia; Kade.Davison@unisa.edu.au; 3 Naturex-DBS LLC, R&D department, South Hackensack, NJ 07606, USA; e.fromentin@naturex-dbs.com

**Keywords:** vascular function, anthocyanins, flow-mediated dilation, vascular reactivity, vascular stiffness, cardiovascular, berries

## Abstract

Anthocyanins are of interest due to their anti-oxidative and vasodilatory properties. Earlier reviews have shown that berries and other anthocyanin rich foods or extracts can improve vascular health, however the effect of anthocyanins on vascular function has not yet been reviewed. To address this gap in the literature, we conducted a systematic review and meta-analysis of randomised-controlled trials examining anthocyanin-rich foods or extracts on measures of vascular reactivity and/or stiffness in adults. Data from 24 studies were pooled as standardized mean difference (SMD) with 95% confidence intervals (CI). Anthocyanin consumption significantly improved flow-mediated dilation (FMD) following acute (SMD: 3.92%, 95% CI: 1.47, 6.38, *p* = 0.002; I^2^ = 91.8%) and chronic supplementation (SMD: 0.84%, 95% CI: 0.55, 1.12, *p* = 0.000; *I*^2^ = 62.5%). Pulse wave velocity was improved following acute supplementation only (SMD: −1.27 m/s, 95% CI: −1.96, −0.58, *p* = 0.000; *I*^2^ = 17.8%). These results support the findings of previous reviews that anthocyanin rich foods or extracts may indeed improve vascular health, particularly with respect to vascular reactivity measured by FMD. More research is required to determine the optimal dosage, and the long-term effects of consumption.

## 1. Introduction

The onset of cardiovascular disease (CVD) is often accompanied by a steady decline in vascular function. A number of biomarkers and methodologies exist to assess vascular health and function. Common biochemical markers include serum concentrations of inflammatory markers (e.g., tumour necrosis factor-α), adhesion molecules (e.g., intercellular adhesion molecule 1), lipids and lipoproteins (total cholesterol, low and high density lipoprotein cholesterol (LDL-C and HDL-C) and triglycerides), oxidised LDL-C, and clotting factors (e.g., Von Willebrand’s factor) [1]. Common non-invasive functional measures include assessments of blood pressure, arterial stiffness and vascular reactivity. At present, the main methods for determining arterial stiffness are pulse wave velocity (PWV), which directly measures point-to-point pulse wave transit time, and pulse wave analysis (PWA), which uses the pulsatile waveform shape to make assumptions about arterial haemodynamics. The pulse wave profile can be obtained using applanation tonometry of the radial or brachial arteries or finger plethysmography using tissue volume changes or infrared detection of flow volume changes. PWA can provide an average measure of overall arterial stiffness based on recordings of peripheral pressure waveforms which are quantified and expressed using a range of different techniques and terms depending on the specific approach or device used [2]. Vascular reactivity can be assessed via peripheral arterial tonometry (PAT), which measures endothelium dependent vasodilation determined by a change in blood flow volume or velocity (via pulse oximetry or laser Doppler imaging (LDI)) typically following a period of partial upper limb occlusion of flow, known as reactive hyperaemia. Flow-mediated dilation (FMD), another measure of vascular reactivity, uses ultrasound to assess the ability of the brachial artery to dilate secondary to reactive hyperaemia as per the previous technique [3,4,5]. The methodological details and associated challenges with each technique are well established [6,7]. FMD is considered the gold-standard non-invasive vascular reactivity measures [7], and because FMD in the brachial artery is closely related to coronary endothelial function, it provides a valid indicator of CVD risk [8,9].

Anthocyanins are polyphenols found in the flavonoid family. They are the red, blue or purple pigments observed in many fruits, vegetables and flowers. In addition to providing vibrant colour, anthocyanins are of interest because of their known bioactivity including potent antioxidant capacity [10] and potential to change markers of health and function of the vascular endothelium. For example, oxidative damage to endothelial cells may interfere with their ability to produce nitric oxide (NO), a powerful vasodilator, thus contributing to endothelial dysfunction [1]. Cell culture studies have demonstrated that the incorporation of anthocyanins into endothelial cells can protect against insult from oxidative stressors [11,12]. Anthocyanins also increase the expression of endothelial nitric oxide synthase, an enzyme that generates NO [13,14]. More recent research suggests that anthocyanins regulate a number of complex immune and inflammatory signalling pathways involved in maintaining vascular health [15]. This suggests that anthocyanin consumption may improve vascular function in humans. 

Numerous intervention studies have evaluated the effect of isolated anthocyanins, anthocyanin-rich foods or their extracts on CVD risk factors, including some markers for vascular health. In a recent meta-analysis of 22 randomised-controlled trials (RCTs), Huang and colleagues [16] demonstrated that anthocyanin-rich berry consumption (2–12 weeks duration) significantly lowered body mass index (BMI) (*p* < 0.00001), LDL-C (*p* = 0.003), fasting glucose (*p* = 0.004), haemoglobin A1c (*p* = 0.04) and tumour necrosis factor-α (*p* = 0.04) in both healthy and metabolically compromised participants. Similarly, a systematic review of 12 RCTs reported that consumption of purified anthocyanins and anthocyanin rich extracts (primarily from berries; 7.35–640 mg anthocyanins/day) improved blood pressure and LDL-C in post-myocardial infarction and hyperlipidemic individuals, respectively [17]. However, the effect of anthocyanins on functional measures of vascular health, other than blood pressure, has not been systematically reviewed. Therefore, the aim of the present study was to conduct a systematic review and meta-analysis of RCTs to evaluate the effect of anthocyanins and anthocyanin rich foods and extracts on vascular function (i.e., arterial stiffness and vascular reactivity) in adults.

## 2. Methods

### 2.1. Protocol and Registration

This systematic review was conducted according to the Preferred Reporting Items for Systematic Reviews and Meta-Analyses (PRISMA) guidelines [18]. The study was registered prospectively on Prospero in May 2016 (registration No. CRD42016037645).

### 2.2. Eligibility Criteria

The eligibility criteria used in the review are summarised in Table 1.

#### 2.2.1. Types of Studies

Randomised-controlled trials that compared anthocyanin-rich foods, anthocyanin-rich extracts or purified anthocyanins against a placebo or control.

#### 2.2.2. Types of Participants

Adult participants aged ≥18 years old.

#### 2.2.3. Types of Intervention

All sources (anthocyanin-rich foods, anthocyanin-rich extracts or purified anthocyanins), doses and durations of anthocyanin intake were considered. Studies included were acute, short-term interventions consisting of a single dose, or chronic, long-term intervention consisting of days, weeks or months of a daily dose. Studies were required to report a quantitative or quantifiable anthocyanin dose; where these data were not reported, information for the investigational product or food was sourced from other published material. If content was not quantified and the product or food did not have a reference value available from other peer-reviewed literature, the study was excluded from analysis. Studies were excluded if they combined anthocyanin-rich food or extracts with other substances, or did not compare anthocyanin-rich food or extract alone to a placebo. Studies evaluating whether anthocyanin-rich foods or extracts could mitigate postprandial effects or other vascular insults (e.g., cigarette smoking) were also excluded.

#### 2.2.4. Comparators

Any comparators (including a placebo or control) that did not contain anthocyanins were eligible for inclusion.

#### 2.2.5. Types of Outcome Measures

Functional measure of vascular health, including assessments of arterial stiffness via PWV, digital volume pulse (DVP), PWA, PAT and Finapres, and vascular reactivity via PAT, LDI and FMD. Studies were excluded if the outcomes comprised only of blood pressure or biochemical markers of vascular function, as these measures have been examined in previous reviews [16,17].

#### 2.2.6. Limiters

No restrictions regarding publication dates were imposed, however, studies were only eligible if they were published in English or a translation was accessible. Only study outcomes published as full papers in peer reviewed scientific journals were included.

### 2.3. Information Sources

Researchers (LF and AMH) searched the following databases: Embase (1980), Medline (1946), Cochrane (1993), CINAHL (Cumulative Index to Nursing and Allied Health, 1982) and Scopus (1960) for relevant articles from inception until 13 June 2017. Further literature searches included manually checking the reference lists of all pertinent articles and reviews for studies missed by the database searches.

### 2.4. Search

Search terms and MeSH/keyword terms were collated into 2 key concepts: anthocyanin rich foods or extracts and vascular function. A review article evaluating high anthocyanin containing foods was used to assist with the development of the anthocyanin concept key words [19]. Relevant key words/terms were also sourced from previous systematic reviews on vascular function. An academic librarian (University of South Australia) reviewed the search strategy prior to any database searches. The search strategy was adapted for each database and can be found in the supplementary information.

The following terms were searched in all databases: ((Anthocyanin* or berry or berries or chokeberry* AND/OR aronia or melanocarpa aubergine* or brinjal* or eggplant* or “solanum melongena*” or “Guinea squash” or “Solanum insanum” or “black currant*” or “Ribes nigrum” or blueberr* or “Vaccinium corymbosum” or “Vaccinium cyanococcus” or “blood orange*” or cherry or cherries or “Cerasus vulgaris” or “Prunus cerasus” or “Prunus avium” or “grape*” or rhubarb or “rheum rhabarbarum” or strawberr* or “fragaria vesca” or “Fragaria ananassa” or blackberr* or raspberr* or rubus glaucus or Rubus fruticosus or plum or plums or “red cabbage*” or “purple cabbage*” or “Brassica oleracea var capitata f rubra” or “red wine” or cranberr* or “vaccinium macrocarpon*” or elderberr* or “sambucus Canadensis” or bilberr* or “vaccinium myrtillus” or whortleberr*) and (coronary circulation” or “vascular stiffness*” or vasodilation or “vascular function*” or “endothelial function*” or “vascular reactivity” or “blood vessel reactivity” or “artery compliance” or “arterial compliance” or “arterial stiffness*” or “small artery elasticity INDEX” or “large artery elasticity INDEX” or saei or laei or “pulse wave” or “augmentation INDEX” or “reflective INDEX” or “beta stiffness INDEX” or “flow mediated dilation” or “flow mediated dilatation” or fmd or “brain circulation*” or “brain blood flow*” or “cerebrovascular reactivit*” or “cerebral blood flow*” or “cerebrovascular circulation” or “pulse pressure” or “coronary artery blood flow” or “coronary arterial flow” or “coronary artery flow” or “coronary blood flow” or “coronary circulation” or “coronary flow” or “heart blood flow” or “Vascular stiffness” or vasodilatation or “vascular endothelium dependent relaxation” or “blood vessel dilatation” or “vascular resistance” or “systemic vascular resistance” or “peripheral resistance” or “arterial pressure wave” or “brain circulation” or “brain blood flow” or “cerebral circulation*” or “cephalic blood flow” or “cerebrum blood flow” or “cerebral bloodflow” or “cerebral blood circulation”)).

### 2.5. Study Selection

Once duplicates were removed, the titles and abstracts of articles were independently screened for eligibility by two investigators (LF and AMH). If consensus was reached, ineligible articles were excluded and eligible articles were moved to the next stage for a full-text review. Articles without sufficient information in the title and abstract were also moved to full text review. All disagreements were resolved by discussion among the research team until a consensus was reached.

### 2.6. Data Collection Process

A data extraction template was developed and pilot tested by 2 authors (LF and AMH) on 5 randomly selected studies. The template was refined according to pilot testing, and any disagreements were resolved by consensus. All data were independently extracted by two investigators (LF and EF), checked for consistency and resolved by consensus.

### 2.7. Data Items

Extracted data included study design, country, sample size, participant characteristics (including mean age, mean BMI, gender, and health status), amount of food/extract and anthocyanins consumed, placebo or control, duration of the intervention, and vascular function outcome of interest.

### 2.8. Quality Assessment

Assessors (LFJ and AMH) independently evaluated the quality of included studies using the 3-category Jadad scoring system [20]. The quality of reporting was determined at the study level, whereby each study was given a score between 1 and 5 based on how well the outlined criteria were met, with a higher score indicating a greater quality. Criteria included reporting randomisation (1 point for mention and 1 point for description and appropriateness of process), blinding (1 point for mention of “double blind” and 1 point for description and appropriateness) and fate of all participants (1 point). Points were removed if the described blinding or randomisation processes insufficiently protected against bias.

### 2.9. Method of Analysis

A descriptive analysis was completed and effect sizes (Cohen’s *d*) for individual studies were determined. Effect sizes were calculated as the difference between treatment and control post-test means, divided by their pooled standard deviations [21], and they were considered small, medium or large based on previous recommendations [22]. Where only standard error of the mean (SEM) was reported, standard deviation was calculated using the following formula:

SD = SEM × square root (*n*), where *n* is the number of participants. (1)

Additionally, meta-analyses were performed using Stata 10.0 (Stata-Corp, College Station, TX, USA). Chronic and acute study data were assessed separately, and in each analysis, data were grouped by vascular function outcome (e.g., FMD, RHI, etc.). Any outcomes involving fewer than two studies were excluded from the meta-analysis. Data from studies that involved multiple anthocyanin doses were averaged. Where acute studies examined multiple time points, only the dose with the peak effect was selected for inclusion in the meta-analysis. Where there were multiple measures from the same outcome (e.g., PWV on left and right sides of the body), these data were combined. Effect sizes were expressed as standardised mean difference (SMD), otherwise known as Cohen’s *d*, and 95% confidence interval (CI).

If not provided by the author, the mean difference (change between baseline and post-test results) was calculated for both intervention and control groups in each single study. The standard deviation of combined means was calculated using the following formula, assuming a correlation coefficient (*R*) = 0.5:

SD = square root [(SDpre-treatment)^2^ + (SDpost-treatment)^2^ − (2R × SDpre-treatment × SDpost-treatment)]. (2)

Within each subgroup, inter-study heterogeneity was measured using the *I*^2^ index, with a value of greater than 50% to indicate significance. Data were pooled using a random-effects model to compensate for the heterogeneity of studies in terms of design, treatment type and duration, and participant characteristics. Forest plots were generated to demonstrate findings. Funnel plots, Begg’s adjusted rank correlation tests and Egger’s regression asymmetry tests were produced to assess likelihood of publication bias.

## 3. Results

### 3.1. Study Selection

**Figure 1:**
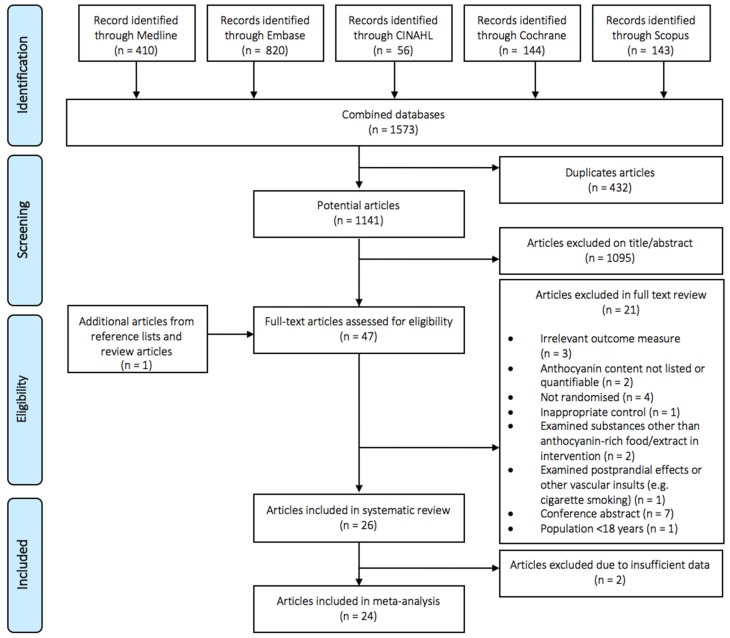
Flow diagram of publication selection.

Figure 1 presents a flow diagram detailing the literature search and selection of studies. A total of 47 publications were identified for a full-text screening, of which 21 were excluded, leaving 26 that were eligible for inclusion in the systematic review and 24 that could be included in the meta-analysis.

### 3.2. Study Characteristics

Study characteristics are summarised in Table 2 and Table 3. Of the 26 eligible publications included in the systematic review [23,24,25,26,27,28,29,30,31,32,33,34,35,36,37,38,39,40,41,42,43,44,45,46,47,48], 29 studies were described: 8 acute and 21 chronic interventions. Two publications reported one chronic and one acute study in the one publication [27,48]. Another publication reported two acute studies in the one publication; one examining time and dose-dependence (three different anthocyanin doses, and four separate time-points) and the other looking at dose-dependence only (five different doses) [41]. The results of all studies (acute and chronic) are reported separately in the tables, irrespective of whether they are combined within a publication. Finally, three publications reported results of high-dose and low/moderate-dose anthocyanin administration [29,34,46]. Studies containing differing doses and/or time points are presented as a single study in the tables and the subsequent tallies.

Studies were predominantly conducted in the United Kingdom (*n* = 9) [31,33,34,36,37,41,42,47] and North America (*n* = 8) [23,26,27,32,38,43,45]. Six studies were completed in Asia (China, *n* = 3; Korea, *n* = 3) [28,29,30,35,48], three in Italy [24,25,40], and one each in Australia [39], Greece [44] and Israel [46]. Of the 29 studies, 15 were parallel [27,28,29,30,31,32,34,35,36,37,38,39,43,45,48] and 14 were crossover designs [23,24,25,26,27,31,33,40,41,42,44,47,48]. 

The number of participants that completed each study ranged from 10 to 146. Seven studies had exclusively male participants [23,25,40,41,42,43], two included postmenopausal females only [32,39], and 19 included both males and females [24,26,27,28,29,30,31,32,34,35,36,37,38,39,44,45,46,47,48]. Most studies (*n* = 19) involved participants in the middle to older age group (mean age ≥ 40 years) [23,24,26,27,28,29,30,31,32,34,35,39,40,43,45,46,48]. Age was not reported in one study [48]. The mean BMI of participants varied, and fell in the healthy (*n* = 13) [25,29,30,31,34,35,36,37,39,41,42,43] and overweight or obese categories (*n* = 13) [23,24,26,27,28,33,34,39,43,45,46,48], while three studies did not report BMI [32,44,47]. Participants were healthy (*n* = 13) [25,31,34,35,36,37,39,41,42,43,44,47], pre-hypertensive/hypertensive (*n* = 5) [29,32,33,38,46], hypercholesterolemic (*n* = 2) [48], possessing other non-specific CVD risk factors (*n* = 4) [24,27,40], and had Metabolic Syndrome (*n* = 4) [23,28,30,45] or CHD (*n* = 1) [26].

Anthocyanins were in the form of a fruit extract in all studies except the two reported by Zhu and colleagues [48], where purified anthocyanins were used. Anthocyanin intakes ranged from 1 to 724 mg/day. Intervention durations ranged from 1 h to 6 h in the acute studies, and one week to six months in the chronic studies. All studies compared the intervention to a placebo or control.

Various measures of vascular function were examined in the eligible studies (Table 2 and Table 3). The relevant outcome measures for arterial stiffness were AI (assessed via all methods including PAT, *n* = 9), PWV (*n* = 10), DVP-SI (*n* = 2) and total peripheral resistance (TPR) assessed via Finapres (*n* = 1). Outcomes measures for vascular reactivity included FMD (*n* = 13), PAT-Reactive Hyperemia Index (PAT-RHI, *n* = 6) and LDI (*n* = 2).

### 3.3. Quality Assessment

**Figure 2:**
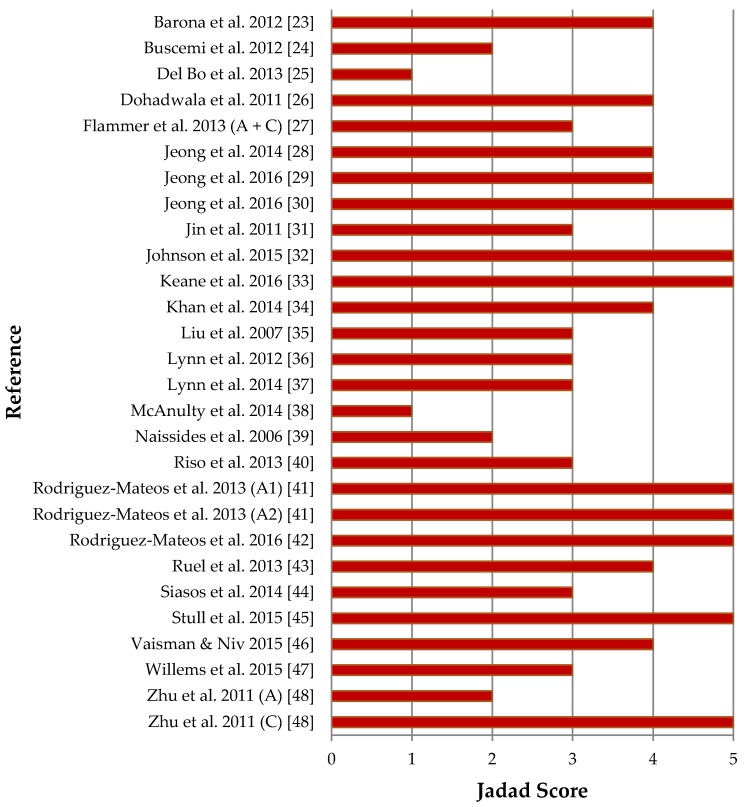
Jadad quality assessment of eligible studies; Abbreviations: A, Acute study; A1, Acute study 1; A2, Acute study 2; C, Chronic study.

Figure 2 details the quality assessment results of publications included in the systematic review. Two publications reported on two studies within the one publication, each involving different participants [41,48]. The scorings for these studies are shown separately in Figure 2. One publication reported outcomes for two studies involving the same participants, and was therefore scored as one study [27]. Quality of reporting was therefore conducted on 28 separate studies. Quality ranged from 1 to 5, with an average quality of 3.5/5. Most of the studies (*n* = 23) had a Jadad score of 3 or greater. All studies were randomised and most featured double-blinding (*n* = 20). Most studies provided an account of participants (*n* = 23). Points were most frequently not attained for describing the method of randomisation (only 12 studies provided a sufficient description) and/or describing the method of blinding (provided by 17 studies). 

### 3.4. Effects of Anthocyanins on Vascular Function

Of the 29 studies from 26 publications included in the systematic review, 18 reported significant improvements in at least one measure of vascular function, whilst 11 reported no improvement. These benefits were observed equally in non-healthy (*n* = 9) and healthy populations (*n* = 9). Vascular function did not deteriorate significantly in any study. 

Data from 24 studies were subsequently included in the meta-analyses. One acute study examining only LDI [31], and a chronic study examining TPR [47] were not incorporated into the meta-analysis as comparable studies including these variables were not available for chronic and acute supplementation studies, respectively. Consequently, only studies reporting on FMD, RHI, PWV and AI were included as these variables were assessed in both acute and chronic studies.

#### 3.4.1. Acute Studies

**Figure 3:**
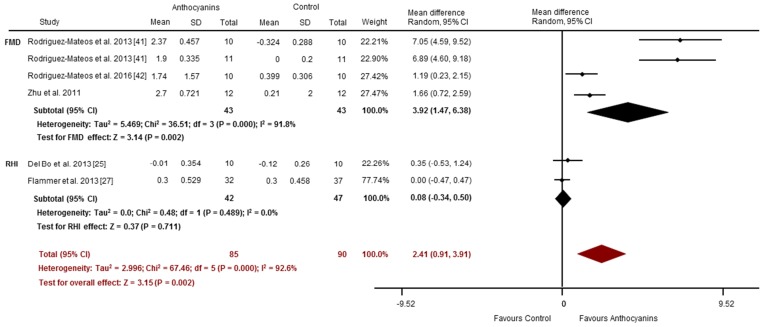
Acute effects of anthocyanins relative to control for vascular reactivity (assessed via FMD and RHI). The forest plot has one line representing each study in the meta-analysis, plotted according to the mean difference (indicated by the black diamond in each line). The horizontal line joins the lower and upper limits of the 95% CI of these effects. The black diamond at the bottom of each graph represents the average effect size for FMD and RHI studies. The red diamond represents the average effect size for all studies assessing vascular reactivity.

**Figure 4:**
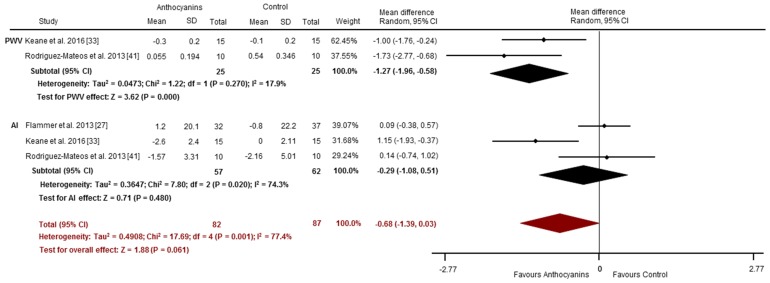
Acute effects of anthocyanins relative to control for vascular stiffness (assessed via PWV and AI). The forest plot has one line representing each study in the meta-analysis, plotted according to the mean difference (indicated by the black diamond in each line). The horizontal line joins the lower and upper limits of the 95% CI of these effects. The black diamond at the bottom of each graph represents the average effect size for PWV and AI studies. The red diamond represents the average effect size for all studies assessing vascular reactivity.

Effects of acute anthocyanin supplementation on vascular reactivity assessed via FMD and RHI were reported in four and two studies, respectively. Compared to placebo-control, acute anthocyanin supplementation significantly improved FMD (SMD: 3.92%, 95% CI: 1.47, 6.38, *p* = 0.002; I^2^ = 91.8%; Figure 3). These studies observed significant increases 1–8 h post consumption of anthocyanin doses between 7 and 724 mg [41,42,48]. No improvements were observed in PAT-RHI (SMD: 0.08, 95% CI: −0.34, 0.50, *p* = 0.71; *I*^2^ = 0%; Figure 3). Collectively, (i.e., the pooling of studies using FMD and/or PAT-RHI) anthocyanins may improve vascular reactivity (Overall SMD 2.41, 95% CI: 0.91, 3.91, *p* = 0.002; *I*^2^ = 92.6%; Figure 3). Arterial stiffness was evaluated via PWV (*n* = 2) [33,41] and AI (*n* = 3) [27,33,41]. PWV was significantly improved following anthocyanin supplementation compared to placebo (SMD: −1.27 m/s, 95% CI: −1.96, −0.58, *p* = 0.000; *I*^2^ = 17.9%; Figure 4). There were no significant changes for AI (SMD: −0.29%, 95% CI: −1.08, 0.51, *p* = 0.48; *I*^2^ = 74.3%; Figure 4). Overall, there was a trend toward an improvement in vascular stiffness (pooled PWV and/or AI studies) with acute anthocyanin supplementation (Overall SMD −0.68, 95% CI: −1.39, 0.03, *p* = 0.061; *I*^2^ = 77.4%; Figure 4).

#### 3.4.2. Chronic Studies

**Figure 5:**
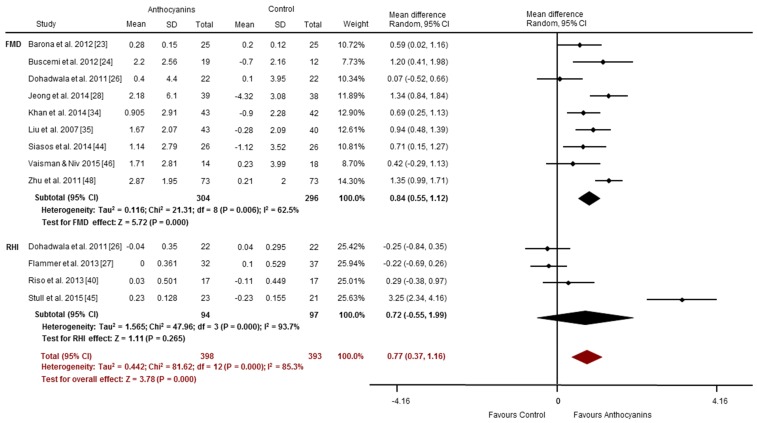
Chronic effects of anthocyanins relative to control for vascular reactivity (assessed via FMD and RHI). The forest plot has one line representing each study in the meta-analysis, plotted according to the mean difference (indicated by the black diamond in each line). The horizontal line joins the lower and upper limits of the 95% CI of these effects. The black diamond at the bottom of each graph represents the average effect size for FMD and RHI studies. The red diamond represents the average effect size for all studies assessing vascular reactivity.

Most of the chronic intervention studies (14 of 20) reported improvements in vascular function [23,24,26,28,30,32,34,35,38,39,44,45,47,48]. These benefits were primarily observed for vascular reactivity assessed via FMD (7 of 9 studies) [23,24,28,34,35,44,48]. Duration of intervention ranged from one week to six months, and used anthocyanin doses of 12 to 320 mg/day. PAT-RHI increased significantly in one of four studies [45]. FMD increased (SMD: 0.84%, 95% CI: 0.55, 1.12, *p* = 0.000, Figure 5), with significant heterogeneity (*I*^2^ = 62.5%). Compared to control, chronic anthocyanin supplementation did not significantly improve RHI (SMD: 0.72, 95% CI: −0.55, 1.99, *p* = 0.265; *I*^2^ = 93.7%; Figure 5). Collectively (i.e., the pooling of studies using FMD and/or PAT-RHI), chronic anthocyanin supplementation may improve vascular reactivity (Overall SMD 0.77, 95% CI: 0.37, 1.16, *p* = 0.000; *I*^2^ = 85.3%; Figure 5).

**Figure 6:**
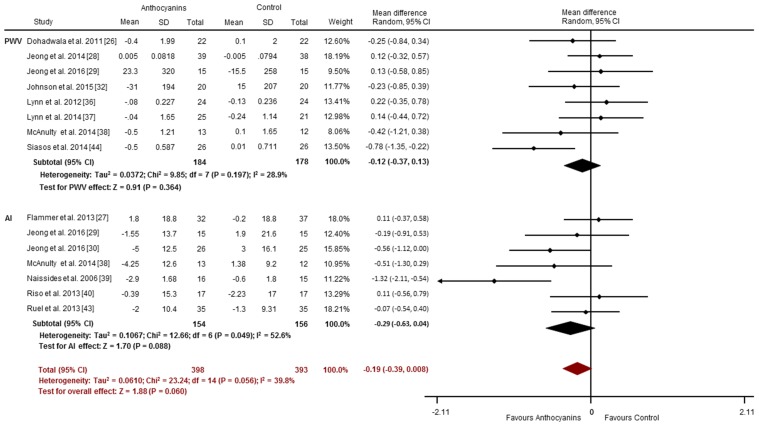
Chronic effects of anthocyanins relative to control for vascular stiffness (assessed via PWV and AI). The forest plot has one line representing each study in the meta-analysis, plotted according to the mean difference (indicated by the black diamond in each line). The horizontal line joins the lower and upper limits of the 95% CI of these effects. The black diamond at the bottom of each graph represents the average effect size for PWV and AI studies. The red diamond represents the average effect size for all studies assessing vascular reactivity.

Less consistent effects were observed for arterial stiffness, with less than half of the studies (6 of 15) reporting improvements in PWV (3 of 8 studies) [26,32,44] or AI (3 of 7 studies) [30,38,39]. Chronic anthocyanin consumption may not improve PWV (SMD: −0.12 m/s, 95% CI: −0.37, 0.13, *p* = 0.364; *I*^2^ = 28.9%; Figure 6), or AI (SMD: −0.29%, 95% CI: −0.63, 0.04, *p* = 0.088; *I*^2^ = 52.6%; Figure 6). Overall, there was a trend toward an improvement in vascular stiffness (pooled PWV and/or AI studies) with chronic anthocyanin supplementation (Overall SMD −0.19, 95% CI: −0.39, 0.008, *p* = 0.060; *I*^2^ = 39.8%; Figure 6).

### 3.5. Publication Bias

Visual inspection of the funnel plots showed symmetry for all outcomes aside from acute studies examining FMD. Begg’s adjusted rank correlation tests revealed non-significant Kendall Tau(s) for all measurements. Egger’s regression asymmetry tests yielded non-significant intercepts for all outcomes, except acute studies examining FMD (*p* = 0.01).

## 4. Discussion

This is the first study to systematically review the effect of anthocyanins rich foods and extracts on functional measures of vascular health. Our meta-analyses showed a statistically significant improvement in vascular reactivity, measured by FMD, resulting from both acute and chronic anthocyanin supplementation. Additionally, this analysis revealed a significant effect on PWV with acute anthocyanin supplementation. We found no significant benefits of acute or chronic supplementation on AI or RHI. However, the pooling of studies assessing vascular stiffness via these techniques (i.e., AI and RHI) suggest that, overall, vascular stiffness may be improved by anthocyanin consumption, subject to confirmation with more data.

These findings are in line with previous reviews that have found a relationship between anthocyanin intake and improvements in vascular function assessed by blood pressure and biochemical markers [16,17], supporting the notion that increasing consumption of anthocyanins may promote vascular health. The majority of RCTs included in this review found significant improvements in vascular reactivity following acute and chronic consumption of anthocyanin-rich foods or extracts, although the effect sizes varied considerably even among studies assessing the same outcomes. These improvements in vascular reactivity were observed in diverse populations with regard to health, BMI status, and age. Reductions in arterial stiffness were less consistent, with none of the acute studies and less than half of the chronic studies reporting significant improvements. There are a number of possible explanations for these outcomes.

Most improvements were observed in vascular reactivity assessed via FMD, which may relate to the reliability of this measure. FMD is considered to be the highest standard of assessing endothelial function in subjects that are in the early, reversible stages of endothelial dysfunction [7]. There are, of course, limitations to the FMD technique, in that the measure is quite variable and highly operator dependent, thus increasing the possibility of a type 2 error [7]. Regardless, FMD and the physiological response of reactive hyperaemia is quite specific for endothelium dependent vasodilation, and is therefore a useful measure to detect changes in this outcome and infer changes about endothelial function more generally [7]. This method is considered to be superior to the other non-invasive techniques, which can be problematic for a number of reasons. For example, it has been suggested that the PAT-derived augmentation index is only partially a NO-dependent measure [53], and that PWA may underestimate either reactivity or arterial stiffness due to the relative influences of central stiffness, peripheral tone, blood pressure and heart rate [54].

Kay et al. [55] suggests that flavonoid consumption is more effective at increasing FMD responses in acute versus chronic studies. Certainly, we found consistently large effect sizes in the acute versus chronic studies; however, we note that the small number of acute studies included in this review limits our conclusions. Overall, our findings support an acute and chronic effect of anthocyanins on FMD. Improvements in chronic studies may better represent a likely improvement in the underlying health or functional status of the vascular tissue. However, there was significant heterogeneity in the FMD results, and some chronic studies failed to demonstrate improvements. Dohadwala and colleagues [26] failed to observe an improvement in vascular reactivity in people with CHD after four-week supplementation with cranberry juice (94 mg/day anthocyanins). It is possible that the effectiveness of anthocyanins is limited in this population as established CVD has been associated with a severely impaired or abnormal endothelial response to vasodilatory stimuli [56,57]. In addition, Vaisman and Niv [46] administered a substantially lower anthocyanin dose (6.7 mg/day for 12 weeks) than other chronic studies reporting a significant improvement in FMD (12 to 320 mg/day). A previous systematic review identified a non-linear dose dependent relationship between FMD responses and intake of certain classes of flavonoids (*R*^2^ ≤ 0.30) [55]. This suggests that, to detect a significant change in vascular function, there is likely a minimum dose that must be consumed.

The majority of chronic studies included in this review were between 1 and 16 weeks, with one study of six-month duration [35]. It is possible that the anthocyanin mechanism of eNOS upregulation may play a role in the acute and chronic effects observed, whereas the antioxidant effect described earlier may not be captured by these studies. Correspondingly, oxidative damage occurs gradually, with endothelial dysfunction and CVD progression thought to occur over many decades. Long-term protection from or reversal of CVD progression may result from nutritional interventions that span over a lifetime, compared to the effects seen in relatively short-term studies [17], as evaluated in this review. Future studies should examine the longer-term effects of supplementation to see if greater improvements in vascular function occur.

Anthocyanin efficacy may be influenced by the chemical composition of extracts, or the process used to manufacture or store them. Anthocyanins are highly unstable and easily degraded by factors such as pH, temperature, light, oxygen exposure, solvents, enzymes, other flavonoids, and proteins, hence it is possible that anthocyanin content may have decreased prior to consumption [58,59]. More thorough reporting of the stability of anthocyanins over time would alleviate this concern, however most studies did not do this. Additionally, only two studies (1 publication) evaluated in this review used purified anthocyanin extracts [48], therefore the majority of test substances would have had a range of other bioactive polyphenols present in them. The polyphenol composition of such extracts was rarely reported, making it difficult to ascertain whether improvements in vascular function can be attributed to anthocyanins alone. This is further complicated by studies reporting synergistic, additive and antagonistic effects on bioactivity when certain polyphenols are combined [35,60,61,62]. It has been suggested that it is a range of flavonoid metabolites that produce the bioactivity rather than the chemicals themselves, and even that different flavonoids may generate common groups of metabolites [63]. This may help explain why different mixes of anthocyanin rich products may have similar effects on vascular function.

There are several other limitations worth mentioning. While the funnel plots did not show asymmetry, Eggers test indicates that there is potential for publication bias in acute studies involving FMD measurements. However, the small number of published papers included in this review makes it challenging to effectively evaluate publication bias. The articles included in this review were limited to those published in English, which may have introduced language bias. Studies where the anthocyanin content was not provided or able to be determined were also excluded, limiting the scope of our evaluation. Most notably, we included a small number of studies, many of which had small sample sizes (*n* < 70) and were heterogeneous with regard to anthocyanin dose, duration and population characteristics. We attempted to reduce the impact of heterogeneity on estimated effect sizes by implementing a random-effects model of analysis. Additionally, some studies were incomplete, failing to report certain population characteristics or outcome data, and necessitating the estimation of anthocyanin intakes based on other publications. Finally, vascular function was assessed using a range of techniques, with varying degrees of accuracy. The Jadad tool used to evaluate study quality did not consider the reliability or validity of these techniques.

While the studies included in this review were on average of moderate quality (>3/5, assessed via the Jadad criteria), not all studies gave an account of participants or adequately described their method of randomisation and blinding. This suggests the possibility of reporting bias, and therefore the results of lower quality studies should be considered with caution. Other systematic reviews have implemented a revised Jadad scale which may be a superior tool given that it assesses additional factors not addressed by the original scale, such as quality of exclusion criteria, the intervention/controls used, and data reporting [64,65]. However, this modified tool did not address our key concerns regarding reliability and validity assessment, and therefore was not applied.

## 5. Conclusions

Our meta-analysis showed that consumption of anthocyanins from foods or extracts significantly improved vascular health. Indeed, acute or chronic anthocyanin consumption resulted in a significant overall increase in measures of vascular reactivity, and significant improvements when assessed specifically by FMD, but not RHI. This analysis included evidence from eight high quality studies (>4/5 Jadad score). Additionally, we found that acute supplementation significantly reduced arterial stiffness as measured by PWV. However, no significant benefits were observed for overall measures of arterial stiffness or AI individually in either acute or chronic studies. Randomised-controlled trials looking at the effect of anthocyanins on vascular reactivity and stiffness have not previously been reviewed; therefore, this meta-analysis is an appropriate addition to the current literature. More research is needed to understand the role of anthocyanins in vascular health, the long-term consequences, and the recommended intake of anthocyanins to assist the reduction of CVD morbidity and mortality. Thus, recommended directions for future research include: (1) dose-dependent studies in specific populations (i.e., varying health conditions) to assist developing dose recommendations; and (2) intervention studies examining a variety of vascular regions, including cerebrovascular vessels.

## Figures and Tables

**Table 1:** Description of the PICOS criteria used to define the research question.

Parameter	Description
P—Population	Healthy or diseased adults
I—Intervention/variable of interest	Anthocyanin-rich foods or extracts or purified anthocyanins
C—Comparator	Control or placebo
O—Outcome	Vascular function as indicated by measures of arterial stiffness or vascular reactivity
S—Study design	Randomised controlled trials

**Table 2:** Acute study characteristics.

Reference	Country	Study Design	No. Randomised	No. Completed	Gender	Mean Age ± SD (Years)	BMI Status, Mean BMI (kg/m^2^)	Health Status	Anthocyanin Source (Food/Extract)	Intake	Anthocyanin (mg/dose)	Control	Duration	Outcomes
Del Bo et al. 2013 [25]	Italy	A, CR	10	10	M	20.8 ± 1.6	Healthy, 22.5	Healthy	Homogenised blueberries	300 g	348	Placebo jelly	1 h	PAT-RHI
Flammer et al. 2013 [27]	USA	A, CR	84	69	M, F	Pl: 51 ± 15.1, I: 45 ± 17.5	Overweight/obese, Pl: 37.2 ± 5.5 I: 27.7 ± 5.9	Endothelial dysfunction, CVD risk, or CVD	Cranberry juice	460 mL	69	Placebo drink	1 h	PAT-RHI PAT-AI
Jin et al. 2011 [31]	UK	A, CR	20	20	M, F	44.6 ± 13.3	Healthy, 23.8 ± 2.5	Healthy	Black currant juice	250 mL	50	Placebo drink	2 h	LDI
Keane et al. 2016 [33]	UK	A, CR	15	15	M	31 ± 9	27.0 ± 3.8	Hypertension	Cherry juice	60 mL	73.5	Control drink	1, 2, 3, 5, 8 h	AI (%) PWV (m/s) LDI DVP-SI (m/s)
Rodriguez-Mateos et al. 2013 [41]	UK	A, CR	11	10	M	27 ± 1.3	Healthy, 25 ± 0.8	Healthy	Blueberry drink	500 mL	310, 517, or 724	Placebo drink	1, 2, 4, 6 h	FMD (%) PWV (m/s) AI (%) DVP-SI (m/s)
Rodriguez-Mateos et al. 2013 [41]	UK	A, CR	11	11	M	27 ± 1.0	Healthy, 22 ± 0.9	Healthy	Blueberry drink	500 mL	129, 258, 310, 517, or 724	Placebo drink	1 h	FMD (%)
Rodriguez-Mateos et al. 2016 [42]	UK	A, CR	10	10	M	24 ± 2	Healthy, 24 ± 2	Healthy	Cranberry juice	450 mL	7, 16, 23, 26, 32	Control drink	1, 2, 4, 6, 8 h	FMD (%)
Zhu et al. 2011 [48]	China	A, CR	12	12	M, F	NR	NR	HC	Purified anthocyanins	320 mg	320	Placebo capsules	1 h	FMD (%)

Abbreviations: A, acute; AI, augmentation index; C, chronic; CR, crossover; CVD, cardiovascular disease; DVP-SI, digital volume pulse—stiffness index; F, females; FMD, flow mediated dilation; HC, hypercholesterolemic; LDI, laser Doppler iontophoresis; M, males; NR, not reported; P, parallel; PAT, peripheral arterial tonometry; Pl, placebo; PWV, pulse wave velocity; RHI, reactive hyperaemia index.

**Table 3:** Chronic study characteristics.

Reference	Country	Study Design	No. Randomised	No. Completed	Gender	Mean Age ± SD (Years)	BMI Status, Mean BMI (kg/m^2^)	Health Status	Anthocyanin Source (Food/Extract)	Intake	Anthocyanin (mg/day)	Control	Duration	Outcomes
Barona et al. 2012 [23]	USA	C, CR	25	24	M	51.3 ± 9.6	Obese, 31.6	Metabolic syndrome	Grape powder	46 g	35	Placebo capsule	30 day	FMD (%)
Buscemi et al. 2012 [24]	Italy	C, CR	21	19	M, F	48 ± 13	Obese, 32.1 ± 4.9	CVD risk factors	Red orange juice	500 mL	36	Placebo drink	7 day	FMD (%)
Dohadwala et al. 2011 [26]	USA	C, CR	47	44	M, F	Pl (1st): 63 ± 9 I (1st): 61 ± 11	Overweight, NR	CHD	Cranberry juice	480 mL	94	Placebo drink	4 week	FMD (%) PAT-RHI crPWV (m/s) cfPWV (m/s)
Flammer et al. 2013 [27]	USA	C, P	84	69	M, F	Pl: 51 ± 15.1 I: 44.8 ± 17.5	Overweight/obese, Pl: 37.2 ± 5.5 I: 27.7 ± 5.9	Endothelial dysfunction, CVD risk factors, CVD	Cranberry juice	460 mL	69	Placebo drink	4 month	PAT-AI PAT-RHI
Jeong et al. 2014 [28]	Korea	C, P	77	77	M, F	Pl: 60.1 ± 9.5 I: 58.0 ± 9.2	Overweight, Pl: 25.1 ± 4.0 I: 26.3 ± 4.3	Metabolic syndrome	Black raspberry powder	750 mg	26 ^a^	Placebo capsule	12 week	PWV (m/s) FMD (%)
Jeong et al. 2016 [29]	Korea	C, P	51	50	M, F	Pl: 60.7 ± 10.4 I: 56.4 ± 9.2	Healthy, Pl: 24.7 ± 3.9 I: 25.9 ± 4.6	Metabolic syndrome	Black raspberry powder	750 mg	26 ^a^	Placebo capsule	12 week	AI (%)
Jeong et al. 2016 [30]	Korea	C, P	45	45	M, F	Pl: 55.9 ± 12.8 MD: 60.2 ± 11.2 HD: 55.5 ± 12.3	Healthy, Pl: 25.8 ± 3.0 MD: 24.5 ± 2.9 HD: 23.5 ± 2.4	Pre-hypertension	MD & HD black raspberry powder	MD: 1500 mgHD: 2500 mg	MD: 52HD: 87 ^a^	Placebo capsule	8 week	AI (%) AI p75 (%) baPWV- (m/s)
Johnson et al. 2015 [32]	USA	C, P	48	40	PF	Pl: 53.7 ± 4.8 I: 59.7 ± 4.6	NR, NR	Pre- and stage 1- hypertension	Blueberry powder	22 g	470	Control powder	8 week	cfPWV (cm/s) baPWV (cm/s)
Khan et al. 2014 [34]	UK	C, P	66	64	M, F	Pl: 51 ± 8 LD: 55 ± 10 HD: 51 ± 11	Overweight, Pl: 28.9 ± 6.5 LD: 28.4 ± 5.4 HD: 29.2 ± 6.9	Healthy	LD or HD black current juice	250 mL	LD: 10 HD: 36	Flavoured water	6 week	FMD (%)
Liu et al. 2007 [35]	China	C, P	103	83	M, F	Pl: 59.8 ± 7.8 I: 58.5 ± 8.6	Healthy, Pl: 23.8 ± 3.6, I: 24.8 ± 3.4	Healthy	Rhubarb capsule	50 mg	12	Placebo capsule	6 month	FMD (%)
Lynn et al. 2012 [36]	UK	C, P	51	48	M, F	C: 36.1 ± 0.92 I: 39.0 ± 1.24	Healthy, C: 25 ± 1.1 I: 25 ± 1.3	Healthy	Pomegranate juice	330 mL	127 ^b^	Lemon drink	4 week	bkPWV (m/s)
Lynn et al. 2014 [37]	UK	C, P	47	46	M, F	C: 37.2 ± 5.8 I: 38.3 ± 6.2	Healthy, C: 24.6 I: 23.5	Healthy	Cherry juice concentrate	30 mL	274	Lemon drink	6 week	bkPWV (m/s)
McAnulty et al. 2014 [38]	USA	C, P	25	NR	M, F	Pl: 39.9 ± 13.4 I: 46.2 ± 11.9	Healthy, Pl: 24.2 ± 3.4 I: 27.8 ± 5.5	Pre-hypertension	Blueberry powder	38 g	625 ^c^	Placebo powder	6 week	cfPWV (m/s) AI (%)
Naissides et al. 2006 [39]	Australia	C, P	45	43	PF	Pl: 59.3 ± 1.4 I: 57.6 ± 1.3	Overweight, Pl: 26.7 ± 1.2 I: 26.3 ± 0.9	Healthy	Dealcoholised red wine	400 mL	283 ^d^	Water	6 week	AI (%)
Riso et al. 2013 [40]	Italy	C, CR	20	18	M	47.8 ± 9.7	Healthy, 24.8 ± 2.6	Healthy (with 1 CVD risk factor)	Blueberry powder	25 g	375	Placebo drink	6 week	PAT-RHI PAT-AI
Ruel et al. 2013 [43]	Canada	C, CR	35	35	M	45 ± 10	Overweight 28.3 ± 2.4	Healthy	Cranberry juice	500 mL	21	Placebo drink	4 week	AI (%) Salbutamol AI (%)
Siasos et al. 2014 [44]	Greece	C, CR	26	NR	M, F	26 ± 5	NR, NR	Healthy, smokers	Concord grape juice	240 mL	71	Grape-fruit juice	7, 14 day	FMD (%) PWV (m/s)
Stull et al. 2015 [45]	USA	C, P	46	44	M, F	PI: 59 ± 2 I: 55 ± 2	Obese, Pl: 36.0 ± 1.1 I: 35.2 ± 0.8	Metabolic syndrome	Blueberry powder	45 g	581	Placebo drink	6 week	PAT-RHI
Vaisman & Niv 2015 [46]	Israel	C, P	50	45	M, F	Pl: 56.4 ± 7.0 MD: 58.5 ± 7.9 HD: 57.6 ± 7.2	Overweight, PL:26.3 ± 4.1 MD: 29.7 ± 3.0 HD: 26.4 ± 3.0	Pre- and stage 1- hypertension	Red grape powder	MD: 200 mg HD: 400 mg	MD: 1.34 HD: 2.68	Placebo	12 week	FMD (%)
Willems, et al. 2015 [47]	UK	C, CR	13	10	M, F	38 ± 8	NR, NR	Healthy	Black currant powder	6 g	139	Placebo drink	7 day	TPR
Zhu et al. 2011 [48]	China	C, P	150	146	M, F	Pl: 70.1 ± 9.8 I: 68.9 ± 8.8	Overweight, Pl: 26.8 ± 2 I: 26.4 ± 2.1	HC	Purified anthocyanins	320 mg	320	Placebo capsule	12 week	FMD (%)

^a, b, c, d^ anthocyanin content estimated from alternate publication (^a^ [49]; ^b^ [50]; ^c^ [51]; ^d^ [52]); Abbreviations: A, acute; AI, augmentation index; ba, brachial-ankle; bk, brachial-knee; cf, carotid-femoral; C, chronic; cr, carotid-radial; CR, crossover; CVD, cardiovascular disease; DVP-SI, digital volume pulse—stiffness index; F, females; FMD, flow mediated dilation; HC, hypercholesterolemic; HD, high dose; I (1st); intervention received first; LD, low dose; LDI, laser Doppler iontophoresis; M, Males; MD, moderate dose; NR, not reported; P, parallel, PAT, peripheral arterial tonometry; Pl, placebo; Pl (1st), placebo received first; PF, postmenopausal females; PWV, pulse wave velocity; RHI, reactive hyperaemia index; TPR, total peripheral resistance.

## Supplementary Materials

The following are available online at www.mdpi.com/2072-6643/9/8/908/s1.
