# Supplementary material for: The Effect of Anthocyanin-Rich Foods or Extracts on Vascular Function in Adults: A Systematic Review and Meta-Analysis of Randomised Controlled Trials
Source: Nutrients. 2017 Aug 20;9(8):908. doi: 10.3390/nu9080908 (PMC5579701; doi:10.3390/nu9080908)
Supplement: Supplementary file 1 [file nutrients-09-00908-s001.pdf]

MEDLINE

1. Anthocyanins/

2. anthocyanin*.mp. [mp=title, abstract, original title, name of substance word, subject heading word, keyword heading word, protocol supplementary concept word, rare disease supplementary concept word, unique identifier]

3. (berry or berries or chokeberr* or aronia or melanocarpa or aubergine* or brinjal* or eggplant* or solanum melongena* or Guinea squash or Solanum insanum or black currant* or Ribes nigrum or blueberr* or Vaccinium corymbosum or Vaccinium cyanococcus or blood orange* or cherry or cherries or Cerasus vulgaris or Prunus cerasus or Prunus avium or grape* or rhubarb or rheum rhabarbarum or strawberr* or fragaria vesca or Fragaria ananassa or blackberr* or raspberr* or rubus glaucus or Rubus fruticosus or plum$1 or red cabbage* or purple cabbage* or Brassica oleracea var capitata f rubra or red wine or cranberr* or vaccinium macrocarpon* or elderberr* or sambucus Canadensis or bilberr* or vaccinium myrtillus or whortleberr*).mp. [mp=title, abstract, original title, name of substance word, subject heading word, keyword heading word, protocol supplementary concept word, rare disease supplementary concept word, unique identifier]

4. Cerebrovascular Circulation/

5. Vascular Stiffness/

6. Solanum melongena/

7. Fragaria/

8. Rubus/

9. VACCINIUM MACROCARPON/

10. SAMBUCUS/

11. VACCINIUM MYRTILLUS/

12. Vasodilation/

13. vascular resistance/

14. Pulse Wave Analysis/

15. (coronary circulation or vascular stiffness* or vasodilation or vascular function* or endothelial function* or vascular reactivity or blood vessel reactivity or artery compliance or arterial compliance or arterial stiffness* or small artery elasticity index or large artery elasticity index or SAEI or LAEI or pulse wave or augmentation index or reflective index or beta stiffness index or flow mediated dilation or flow mediated dilatation or FMD or brain circulation* or brain blood flow* or cerebrovascular reactivit* or cerebral blood flow* or cerebrovascular circulation).mp. [mp=title, abstract, original title, name of substance word, subject heading word, keyword heading word, protocol supplementary concept word, rare disease supplementary concept word, unique identifier]

16. 1 or 2 or 3 or 6 or 7 or 8 or 9 or 10 or 11

17. 4 or 5 or 12 or 13 or 14 or 15

18. 16 and 17

COCHRANE

#1 anthocyanin* or berry or berries or chokeberr* or aronia or melanocarpa or aubergine* or brinjal* or eggplant* or "solanum melongena*" or "Guinea squash" or "Solanum insanum" or "black currant*" or "Ribes nigrum" or blueberr* or "Vaccinium corymbosum" or "Vaccinium cyanococcus" or "blood orange*" or cherry or

cherries or "Cerasus vulgaris" or "Prunus cerasus" or "Prunus avium" or "grape*" or rhubarb or "rheum rhabarbarum" or strawberr* or "fragaria vesca" or "Fragaria ananassa" or blackberr* or raspberr* or rubus glaucus or Rubus fruticosus or plum or plums or "red cabbage*" or "purple cabbage*" or "Brassica oleracea var capitata f rubra" or "red wine" or cranberr* or "vaccinium macrocarpon*" or elderberr* or "sambucus Canadensis" or bilberr* or "vaccinium myrtillus" or whortleberr*

#2 MeSH descriptor: [Anthocyanins] this term only
#3 MeSH descriptor: [Solanum melongena] this term only
#4 MeSH descriptor: [Fragaria] this term only
#5 MeSH descriptor: [Rubus] this term only
#6 MeSH descriptor: [Vaccinium macrocarpon] this term only
#7 MeSH descriptor: [Sambucus] this term only
#8 MeSH descriptor: [Vaccinium myrtillus] this term only
#9 MeSH descriptor: [Blood Pressure] this term only
#10 MeSH descriptor: [Cerebrovascular Circulation] this term only
#11 MeSH descriptor: [Vascular Stiffness] this term only
#12 MeSH descriptor: [Vasodilation] this term only
#13 MeSH descriptor: [Vascular Resistance] this term only
#14 MeSH descriptor: [Pulse Wave Analysis] this term only
#15 "blood pressure" or "coronary circulation" or "vascular stiffness*" or vasodilation or "vascular function*" or "endothelial function*" or "vascular reactivity" or "blood vessel reactivity" or "artery compliance" or "arterial compliance" or "arterial stiffness*" or "small artery elasticity index" or "large artery elasticity index" or SAEI or LAEI or "pulse wave" or "augmentation index" or "reflective index" or "beta stiffness index" or "flow mediated dilation" or "flow mediated dilatation" or FMD or "brain circulation*" or "brain blood flow*" or "cerebrovascular reactivit*" or "cerebral blood flow*" or "cerebrovascular circulation"
#16 #1 or #2 or #3 or #4 or #5 or #6 or #7 or #8
#17 #9 or #10 or #11 or #12 or #13 or #14 or #15
#18 #16 and #17

<u>EMBASE</u>

1. Anthocyanins/

2. anthocyanin*.mp. [mp=title, abstract, original title, name of substance word, subject heading word, keyword heading word, protocol supplementary concept word, rare disease supplementary concept word, unique identifier]

3. (berry or berries or aubergine* or brinjal* or eggplant* or solanum melongena* or Guinea squash or Solanum insanum or black currant* or Ribes nigrum or blueberr* or Vaccinium corymbosum or Vaccinium cyanococcus or blood orange* or cherry or cherries or Cerasus vulgaris or Prunus cerasus or Prunus avium or black grape* or rhubarb or rheum rhabarbarum or strawberr* or fragaria vesca or Fragaria ananassa or blackberr* or raspberr* or rubus glaucus or Rubus fruticosus or plum$1 or red cabbage* or purple cabbage* or Brassica oleracea var capitata f rubra or red wine or cranberr* or vaccinium macrocarpon* or elderberr* or sambucus Canadensis or bilberr* or vaccinium myrtillus or whortleberr*).mp. [mp=title, abstract, original title, name of substance word, subject heading word, keyword heading word, protocol supplementary concept word, rare disease supplementary concept word, unique identifier]

4. Blood Pressure/

5. Cerebrovascular Circulation/

6. Vascular Stiffness/

7. Solanum melongena/

8. Fragaria/

9. Rubus/

10. VACCINIUM MACROCARPON/

11. SAMBUCUS/

12. VACCINIUM MYRTILLUS/

13. Vasodilation/

14. vascular resistance/

15. Pulse Wave Analysis/

16. (blood pressure or coronary circulation or vascular stiffness* or vasodilation or vascular function* or endothelial function* or vascular reactivity or arterial compliance or arterial stiffness* or small artery elasticity index or large artery elasticity index or SAEI or LAEI or pulse wave velocity or pulse wave analysis or augmentation index or reflective index or beta stiffness index or flow mediated dilation or flow mediated dilatation or FMD or cerebrovascular reactivity or cerebral blood flow* or cerebrovascular circulation).mp. [mp=title, abstract, original title, name of substance word, subject heading word, keyword heading word, protocol supplementary concept word, rare disease supplementary concept word, unique identifier]

17. 4 or 5 or 6 or 13 or 14 or 15 or 16

<u>SCOPUS</u>

1. Anthocyanin* or berry or berries or chokeberry* or aronia or melanocarpa aubergine* or brinjal* or eggplant* or "solanum melongena*" or "Guinea squash" or "Solanum insanum" or "black currant*" or "Ribes nigrum" or blueberr* or "Vaccinium corymbosum" or "Vaccinium cyanococcus" or "blood orange*" or cherry or cherries or "Cerasus vulgaris" or "Prunus cerasus" or "Prunus avium" or "grape*" or rhubarb or "rheum rhabarbarum" or strawberr* or "fragaria vesca" or "Fragaria ananassa" or blackberr* or raspberr* or rubus glaucus or Rubus fruticosus or plum or plums or "red cabbage*" or "purple cabbage*" or "Brassica oleracea var capitata f rubra" or "red wine" or cranberr* or "vaccinium macrocarpon*" or elderberr* or "sambucus Canadensis" or bilberr* or "vaccinium myrtillus" or whortleberr*

2. "coronary circulation" OR "vascular stiffness*" OR vasodilation OR "vascular function*" OR "endothelial function*" OR "vascular reactivity" OR "blood vessel reactivity" OR "artery compliance" OR "arterial compliance" OR "arterial stiffness*" OR "small artery elasticity INDEX" OR "large artery elasticity INDEX" OR saei OR laei OR "pulse wave" OR "augmentation INDEX" OR "reflective INDEX" OR "beta stiffness INDEX" OR "flow mediated dilation" OR "flow mediated dilatation" OR fmd OR "brain circulation*" OR "brain blood flow*" OR "cerebrovascular reactivit*" OR "cerebral blood flow*" OR "cerebrovascular circulation" OR "pulse pressure" OR "coronary artery blood flow" OR "coronary arterial flow" OR "coronary artery flow" OR "coronary blood flow" OR "coronary circulation" OR "coronary flow" OR "heart blood flow" OR "Vascular stiffness" OR vasodilatation OR "vascular endothelium dependent relaxation" OR "blood vessel dilatation" OR "vascular resistance" OR "systemic vascular resistance" OR "peripheral resistance" OR "arterial pressure wave" OR "brain circulation" OR "brain blood flow" OR "cerebral circulation*" OR "cephalic blood flow" OR "cerebrum blood flow" OR "cerebral bloodflow" OR "cerebral blood circulation"

3. 1 AND 2

<u>CINAHL</u>

S25	((S3 OR S4 OR S5 OR S7 OR S8 OR S9 OR S10 OR S11 OR S12 OR S13 OR S15 OR S16) AND (S17 OR S18 OR S20 OR S21 OR S22)) AND (S23 AND S24)	Expanders - Apply related words<br>Search modes - Boolean/Phrase
S24	(S3 OR S4 OR S5 OR S7 OR S8 OR S9 OR S10 OR S11 OR S12 OR S13 OR S15 OR S16) AND (S17 OR S18 OR S20 OR S21 OR S22)	Expanders - Apply related words<br>Search modes - Boolean/Phrase
S23	S3 OR S4 OR S5 OR S7 OR S8 OR S9 OR S10 OR S11 OR S12 OR S13 OR S15 OR S16	Expanders - Apply related words<br>Search modes - Boolean/Phrase

S22	"coronary circulation" OR "vascular stiffness*" OR vasodilation OR "vascular function*" OR "endothelial function*" OR "vascular reactivity" OR "blood vessel reactivity" OR "artery compliance" OR "arterial compliance" OR "arterial stiffness*" OR "small artery elasticity INDEX" OR "large artery elasticity INDEX" OR saei OR laei OR "pulse wave" OR "augmentation INDEX" OR "reflective INDEX" OR "beta stiffness INDEX" OR "flow mediated dilation" OR "flow mediated dilatation" OR fmd OR "brain circulation*" OR "brain blood flow*" OR "cerebrovascular reactivit*" OR "cerebral blood flow*" OR "cerebrovascular circulation" OR diastolic OR systolic OR "pulse pressure" OR "coronary artery blood flow" OR "coronary arterial flow" OR "coronary artery flow" OR "coronary blood flow" OR "coronary circulation" OR "coronary flow" OR "heart blood flow" OR "Vascular stiffness" OR vasodilatation OR "vascular endothelium dependent relaxation" OR "blood vessel dilatation" OR "vascular resistance" OR "systemic vascular resistance" OR "peripheral resistance" OR "arterial pressure wave" OR "brain circulation" OR "brain blood flow" OR "cerebral circulation*" OR "cephalic blood flow" OR "cerebrum blood flow" OR "cerebral bloodflow" OR "cerebral blood circulation"	Expanders - Apply related words<br>Search modes - Boolean/Phrase
S21	(MH "Vasodilation") OR (MH "Vascular Resistance")	Expanders - Apply related words<br>Search modes - Boolean/Phrase
S20	(MH "Cerebrovascular Circulation") OR (MH "Blood Flow Velocity")	Expanders - Apply related words<br>Search modes - Boolean/Phrase
S19	(MH "Cerebrovascular Circulation")	Expanders - Apply related words<br>Search modes - Boolean/Phrase
S18	(MH "Coronary Circulation")	Expanders - Apply related words<br>Search modes - Boolean/Phrase
S17		Expanders - Apply related words<br>Search modes - Boolean/Phrase
S16	Brinjal or aubergine or "guinea squash" or "solanum insanum" or "black currant" or "ribes nigrum" or "Vaccinium corymbosum" or "Vaccinium cyanococcus" or cherry or cherries or "Cerasus vulgaris" or "Prunus cerasus" or "Prunus avium" or "black grape*" or "rheum rhabarbarum" or strawberries or "fragaria vesca" or "Fragaria ananassa" or blackberr* or raspberries or rubus glaucus or Rubus fruticosus or plum or plums or "red cabbage*" or "purple cabbage*" or "Brassica oleracea var capitata f rubra" or "red wine" or cranberr* or "vaccinium macrocarpon*" or elderberr* or "sambucus Canadensis" or bilberr* or "vaccinium myrtillus" or whortleberr*	Expanders - Apply related words<br>Search modes - Boolean/Phrase
S15	"plum" OR (MH "Cranberry") OR "cranberry" OR (MH "Cranberry Juice")	Expanders - Apply related words<br>Search modes - Boolean/Phrase
S14	plum	Expanders - Apply related words<br>Search modes - Boolean/Phrase

S13	(MH "Raspberry") OR "raspberry"	Expanders - Apply related words<br>Search modes - Boolean/Phrase
S12	(MH "Strawberry") OR "strawberry"	Expanders - Apply related words<br>Search modes - Boolean/Phrase
S11	(MH "Rhubarb") OR "rhubarb"	Expanders - Apply related words<br>Search modes - Boolean/Phrase
S10	"blood orange" OR (MH "Sour Cherry") OR (MH "Cherries")	Expanders - Apply related words<br>Search modes - Boolean/Phrase
S9	(MH "Wild Cherry") OR "black currant"	Expanders - Apply related words<br>Search modes - Boolean/Phrase
S8	"solanum melongena"	Expanders - Apply related words<br>Search modes - Boolean/Phrase
S7	"eggplant"	Expanders - Apply related words<br>Search modes - Boolean/Phrase
S6	eggplant	Expanders - Apply related words<br>Search modes - Boolean/Phrase
S5	"aubergine"	Expanders - Apply related words<br>Search modes - Boolean/Phrase
S4	(MH "Berries") OR "berries" OR (MH "Blackberry") OR (MH "Elderberry") OR (MH "Blueberry") OR (MH "Bilberry")	Expanders - Apply related words<br>Search modes - Boolean/Phrase
S3	"anthocyanin"	
